# Age and Sex Differences in Adverse Events Associated With Antipsychotics: An Analysis of the FDA Adverse Events Database

**DOI:** 10.1002/gps.70142

**Published:** 2025-08-15

**Authors:** Tabea Ramin, Jens‐Uwe Peter, Michael Schneider, Volker Dahling, Oliver Zolk

**Affiliations:** ^1^ Institute for Clinical Pharmacology Immanuel Hospital Rüdersdorf Brandenburg Medical School Theodor Fontane Rüdersdorf Germany; ^2^ Center for Mental Health Immanuel Hospital Rüdersdorf Brandenburg Medical School Theodor Fontane Rüdersdorf Germany; ^3^ Faculty of Health Sciences Joint Faculty of the University of Potsdam the Brandenburg Medical School Theodor Fontane and the Brandenburg University of Technology Cottbus—Senftenberg Neuruppin Germany

**Keywords:** adverse events, antipsychotic drugs, geriatric psychiatry, older adults, schizophrenia

## Abstract

**Objectives:**

While the risks of antipsychotics in older adults are well recognized, clinical trials often exclude frail older patients, have short follow‐up periods, and provide limited comparative data on specific drugs. This study aimed to explore age‐related differences in the adverse effects of six commonly prescribed antipsychotics using a pharmacovigilance database, with additional analysis of sex‐based variations.

**Methods:**

We analyzed adverse event (AE) reports associated with aripiprazole, clozapine, olanzapine, quetiapine, risperidone, and haloperidol from the FDA Adverse Event Reporting System (FAERS) database between Q4 2003 and Q2 2024. We utilized Standardized MedDRA Queries (SMQs) and self‐defined queries to categorize 18 groups of AEs. Adjusted logistic regression was employed to calculate adjusted reporting odds ratios (aRORs) with 95% confidence intervals (CIs).

**Results:**

Our analysis revealed substance‐specific differences in AE profiles. Risperidone had the highest aROR for hyperprolactinemia (aROR 212, 95% CI 203–221), haloperidol for dystonia (aROR 46, 95% CI 41–51), and aripiprazole for akathisia (aROR 45, 95% CI 42–49). Patients aged 65 and older generally demonstrated a higher likelihood of experiencing cardiac, extrapyramidal motor, and sedative AEs compared to those under 65, with few exceptions across the drugs investigated. In contrast, younger patients showed higher odds for metabolic AEs, including dyslipidemia and hyperglycemia (associated with olanzapine and quetiapine), as well as weight gain (with olanzapine, quetiapine, risperidone, and haloperidol). With few exceptions, women generally showed higher reporting odds of adverse events. Sex‐related differences were especially pronounced for hyperprolactinemia, with 4.7–8.0 times higher reporting odds in women for aripiprazole, olanzapine, quetiapine, and haloperidol—except for risperidone, where a post‐2014 rise in male reports led to higher odds in men. Risperidone was also associated with increased reporting odds of weight gain in men. Additionally, aripiprazole and olanzapine showed 3 to 6 times higher reporting odds for anticholinergic syndrome in men compared to women.

**Conclusions:**

It is essential to consider both age and sex in treatment decisions to optimize the efficacy and tolerability of antipsychotic therapy.

## Summary

- Analysis of adverse event (AE) data for the six most common antipsychotics highlights the influence of age and sex on their side effect profiles.
- Older patients were more prone to cardiac, extrapyramidal motor, and sedative AEs, while younger patients experienced more metabolic AEs, such as dyslipidemia, hyperglycemia, and weight gain.
- Women were equally or more likely than men to experience side effects.

## 1 Introduction

The global population is aging rapidly, with individuals aged 60 and older projected to make up 22% of the population by 2050, up from 12% in 2015 [1, 2]. According to the World Health Organization, 20% of this group experiences mental or neurological disorders, highlighting the need for improved mental health care for older adults [1, 3].

Antipsychotics are widely prescribed to older adults for psychiatric conditions like schizophrenia, bipolar disorder, and depression with psychotic features, as well as off‐label for age‐related issues such as delirium, dementia‐related psychosis, and behavioral and psychological symptoms of dementia (BPSD). Approved uses include brexpiprazole for agitation in Alzheimer's dementia (FDA) [4] and risperidone in Europe for short‐term treatment of persistent aggression in moderate to severe Alzheimer's dementia when non‐pharmacological approaches fail.

Evidence on the safety and efficacy of antipsychotics primarily comes from clinical trials conducted for drug approval. However, these trials often exclude older adults with comorbidities, polypharmacy, or frailty, reducing their applicability to this population [5]. As a result, treatment guidelines, such as those for schizophrenia, lack specific recommendations on the safe use of antipsychotics in older adults [6]. While it is generally known that older patients have distinct pharmacokinetics and pharmacodynamics, as well as increased susceptibility to adverse drug events due to age‐related physiological decline, gaps in trial data leave these issues insufficiently addressed.

One exception is the randomized, placebo‐controlled trials conducted specifically in older patients with dementia to evaluate atypical antipsychotics for behavioral symptoms. In 2005, a meta‐analysis of 17 studies revealed increased mortality risks, leading to updated product warnings [7]. Similar warnings for conventional antipsychotics were initially absent due to limited data, but observational studies later associated both first‐ and second‐generation antipsychotics with increased risks of stroke, venous thromboembolism, myocardial infarction, fractures, pneumonia, acute kidney injury, and mortality in older adults [8, 9, 10].

The American Geriatrics Society's 2023 Beers Criteria advises against antipsychotic use in older adults except for specific conditions such as schizophrenia, bipolar disorder, or Parkinson's disease psychosis but does not specify preferred drugs [11]. Germany's PRISCUS 2.0 list also flags several antipsychotics as potentially inappropriate, particularly at high doses or prolonged use [12]. Interestingly, observational studies indicate that adverse outcomes are most pronounced shortly after treatment initiation rather than during extended use [10]. This highlights the need for better data on compound‐specific side effect profiles to refine antipsychotic prescribing guidelines for older patients. Controlled trial data in this population are limited, and observational studies provide only partial insights. To address this evidence gap, we turned to an alternative approach: analyzing age‐related risk profiles of the six most commonly prescribed antipsychotics using the FDA Adverse Event Reporting System (FAERS), a large pharmacovigilance database.

FAERS compiles reports from clinical trial monitoring and spontaneous adverse reaction reports from healthcare professionals, patients, and manufacturers, offering a broad view of adverse events and identifying rare or long‐term side effects often missed in clinical trials. We also incorporated sex as a factor in our analysis, recognizing that hormonal, metabolic, and pharmacokinetic differences between men and women can influence side effect profiles.

## 2 Materials and Methods

We analyzed FAERS adverse event reports from Q4 2003 to Q3 2024 for 6 commonly prescribed antipsychotics: aripiprazole, clozapine, olanzapine, quetiapine, risperidone, and haloperidol. These drugs were selected based on high prescription rates [13] and sufficient case numbers in FAERS, which included mandatory data on age and sex. Only cases where the drug was classified as the primary suspect (i.e., the most likely cause of the adverse event) were included.

Adverse events in FAERS are coded using Medical Dictionary for Regulatory Activities (MedDRA) terminology. We used standardized medical queries (SMQs), validated sets of MedDRA terms, to analyze drug‐specific adverse event signals. Included SMQs were accidents and injuries, agranulocytosis, anticholinergic syndrome, dyslipidemia, akathisia, dyskinesia, dystonia, Parkinson‐like events, neuroleptic malignant syndrome (NMS), hyperglycemia/diabetes, sexual dysfunction, suicide/self‐injury, and torsade de pointes (TdP)/QT prolongation, applying “narrow” SMQ definitions for higher specificity.

For side effects without predefined SMQs (e.g., sedation, weight gain, cognitive impairment, syndrome of inappropriate antidiuretic hormone secretion (SIADH)/hyponatremia, and hyperprolactinemia), we created custom queries using relevant MedDRA terms. A complete list of predefined and custom SMQs is provided in Table 1. To ensure sufficient statistical power and interpretability, drugs were included in the analysis only if there were at least three reported cases of each SMQ.

**TABLE 1:** Side effects of interest with its MedDRA SMQ and preferred term (PT) names and codes.

SMQ of interest	MedDRA SMQs		PTs used for self‐defined queries	
	SMQ name	SMQ code	PT name	PT code
Fall	Accidents and injuries	20000135		
Agranulocytosis	Agranulocytosis	20000023		
Anticholinergic syndrome	Anticholinergic syndrome	20000048		
Metabolic disorders	Dyslipidaemia	20000026		
	Hyperglycaemia/new onset diabetes mellitus	20000041		
Weight gain	Self‐defined		Abnormal weight gain	10000188
			Obesity	10029883
			Central obesity	10065941
			Overweight	10033307
			Weight increased	10047899
			Body mass index increased	10005897
Extrapyramidal motor system disorders	Akathisia	20000096		
	Dyskinesia	20000097		
	Dystonia	20000098		
	Parkinson‐like events	20000099		
	Neuroleptic malignant syndrome	20000044		
Sexual dysfunction	Sexual dysfunction	20000238		
TdP/QT prolongation	Torsade de pointes/QT prolongation	20000001		
Suicide	Suicide/self‐injury	20000037		
Sedation	Self‐defined		Sedation	10039897
			Somnolence	10041349
Cognitive impairment	Self‐defined		Cognitive disorder	10057668
			Memory impairment	10027175
			Amnesia	10001949
			Confusional state	10010305
			Dementia	10012267
			Disturbance in attention	10013496
			Disorientation	10013395
			Speech disorder	10041466
			Disorganised speech	10076227
			Aphasia	10002948
			Delirium	10012218
SIADH/Hyponatraemia	Self‐defined		Blood sodium decreased	10005802
			Hyponatraemia	10021036
			Inappropriate antidiuretic hormone secretion	10053198
			Hyponatraemic syndrome	10021037
			Hyponatraemic encephalopathy	10066151
			Hyponatraemic seizure	10073183
			Hyponatraemic coma	10075865
Hyperprolactinaemia	Self‐defined		Gynecomastia	10018800
			Blood prolactin abnormal	10005778
			Blood prolactin increased	10005780
			Galactorrhea	10017600
			Hyperprolactinaemia	10020737

Abbreviations: PT, preferred term; SMQ, standardized MedDRA query.

Duplicate cases were removed per FDA recommendations, and the remaining cases were grouped by sex and age (< 65 or ≥ 65 years). We considered finer stratification of age groups; however, due to limitations in available sample sizes and event frequencies within these subgroups, such stratification was not statistically feasible for the present analysis.

For each antipsychotic–SMQ pair, we extracted a 2 × 2 contingency table from the FAERS database as follows.

‒ Cell A: Number of reports including both the antipsychotic of interest and the SMQ of interest.
‒ Cell B: Number of reports including the antipsychotic of interest but not the SMQ of interest.
‒ Cell C: Number of reports including the SMQ of interest but not the antipsychotic of interest.
‒ Cell D: Number of reports including neither the antipsychotic of interest nor the SMQ of interest.

Using this table, the crude Reporting Odds Ratio (ROR) was calculated as (A/B)/(C/D). To derive adjusted RORs (aRORs), we performed binary multivariable logistic regression analyses with the presence of the SMQ as the dependent variable and drug exposure (yes/no) as the primary independent variable. The models were adjusted for sex or age group, as appropriate. Importantly, these comparisons were made relative to the entire FAERS database, not limited to the other antipsychotics included in our study [14]. Risk signals were defined as ROR ≥ 2, Wald chi‐square *p*‐value < 0.05, and ≥ 3 reported cases.

We also calculated sex‐adjusted RORs and 95% CIs for each age group and compared aRORs between age groups using Z statistics. Similarly, we calculated sex‐specific RORs adjusted for age groups and compared aRORs between sexes using Z statistics. To account for multiple comparisons, *p*‐values were adjusted using the Bonferroni‐Holm method, with statistical significance set at *p* < 0.05. Data were analyzed using OpenVigil 2.1 [15] and IBM SPSS Statistics 27.

A sensitivity analysis was conducted to assess the robustness of the results. Specifically, we compared the analysis based on “primary suspect” designations in FAERS case reports with an alternative analysis that included all designation types (“primary suspect,” “secondary suspect,” “concomitant,” and “interacting”). For the sensitivity analysis, RORs for each drug–SMQ pair were calculated separately for each age and sex subgroup.

## 3 Results

### 3.1 Disproportionality Analysis

Only FAERS reports with age and sex data were included. Report counts ranged from 8 (anticholinergic syndrome for aripiprazole) to 6283 (hyperglycemia for quetiapine). Table 2 details the total reports, along with the percentages of female and older adults (≥ 65 years), for each antipsychotic and adverse event SMQ.

**TABLE 2:** Number of cases and the percentage of female and older adults (≥ 65 years).

	Clozapine			Aripiprazole			Olanzapine			Quetiapine			Risperidone			Haloperidol		
SMQ	N	Female (%)	≥ 65 years (%)	N	Female (%)	≥ 65 years (%)	N	Female (%)	≥ 65 years (%)	N	Female (%)	≥ 65 years (%)	N	Female (%)	≥ 65 years (%)	N	Female (%)	≥ 65 years (%)
Accidents/injuries	1052	38.3	24.5	810	57.4	17.4	872	47.0	36.1	2199	59.5	21.6	609	47.6	39.2	147	51.0	34.7
Agranulocytosis	921	36.3	13.5	93	51.6	28.0	290	46.6	28.3	212	50.0	20.8	110	48.2	32.7	18	22.2	38.9
Anticholinergic syndrome	21	42.9	4.8	8	12.5	0.0	46	23.9	15.2	77	79.2	26.0	36	36.1	13.9	10	20.0	10.0
Dyslipidaemia	341	32.0	4.7	322	42.5	4.3	1182	42.1	4.7	1583	54.1	2.3	236	28.4	5.5	25	32.0	4.0
Weight gain	487	40.5	3.5	1817	60.8	5.0	2366	51.8	5.4	2644	65.1	3.8	2553	14.7	1.3	538	49.4	3.7
Hyperglycaemia	693	46.2	8.4	1037	58.1	8.2	3213	47.5	7.5	6283	58.3	5.0	596	38.8	10.6	61	45.9	13.1
Suicide	986	44.5	3.7	1886	57.2	3.1	2170	53.5	5.7	6030	63.7	6.3	1948	50.3	5.5	733	51.6	5.6
Akathisia	67	47.8	7.5	864	52.7	4.1	341	49.6	6.5	276	60.1	10.9	268	41.4	11.6	127	44.1	7.9
Dyskinesia	336	50.6	11.3	1796	60.7	12.7	620	49.8	14.5	1283	52.8	13.7	863	40.3	11.2	337	39.5	15.4
Dystonia	264	55.7	6.8	872	53.9	3.7	478	54.6	10.5	531	54.4	9.4	665	39.1	10.2	397	36.8	5.5
Parkinson‐like events	314	38.2	47.8	797	54.2	23.8	610	39.8	34.8	720	51.9	39.0	634	40.5	35.2	378	42.1	28.6
NMS	393	25.2	7.9	592	39.9	12.7	915	36.9	15.6	1146	45.6	28.6	669	41.3	15.8	422	37.7	20.4
Sexual dysfunction	144	50.7	0.0	426	39.4	3.3	317	37.9	2.5	440	30.0	4.1	286	20.3	3.1	35	11.4	2.9
TdP/QT prolongation	325	43.7	5.8	202	64.9	8.9	504	52.2	26.2	995	63.1	18.9	273	54.6	28.2	140	42.1	32.1
Sedation	1116	43.5	16.9	1222	55.4	6.0	1888	44.2	14.7	2973	61.8	18.8	1185	35.3	18.1	331	44.4	23.0
Cognition	999	39.5	25.3	1283	54.6	13.7	1958	41.7	26.0	2752	58.6	28.7	1091	44.3	35.3	424	45.3	33.3
SIADH/Hyponataemia	152	38.2	11.8	167	58.1	34.1	395	38.2	19.0	296	63.2	36.1	241	49.4	34.0	64	53.1	43.8
Hyperprolactinaemia	169	68.6	0.0	424	73.8	0.5	416	64.9	2.4	322	70.5	1.2	4480	12.3	0.7	71	52.1	0.0

Abbreviations: NMS, neuroleptic malignant syndrome; SIADH, syndrome of inappropriate antidiuretic hormone secretion; TdP, torsade de pointes.

**FIGURE 1:**
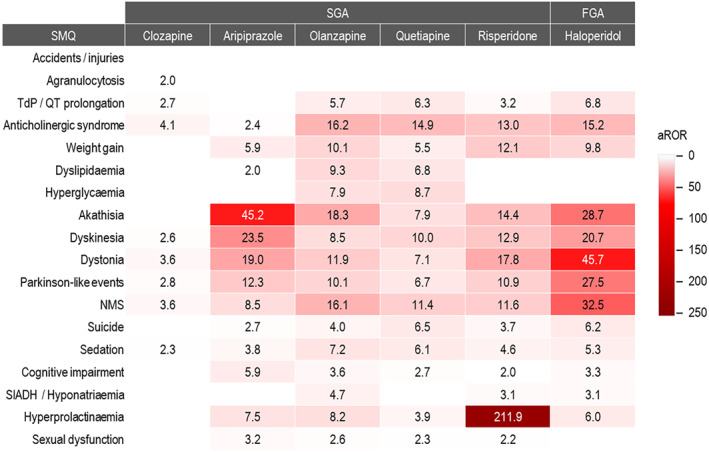
Heat map displaying reporting odds ratios (RORs) adjusted for age group and sex. Cells are left blank if the adjusted ROR is < 2 and/or the Bonferroni‐Holm corrected *p*‐value is ≥ 0.05. FGA, first‐generation antipsychotic; NMS, neuroleptic malignant syndrome; SGA, second‐generation antipsychotic; SIADH, syndrome of inappropriate antidiuretic hormone secretion; TdP, torsade de pointes.

Figure 1 displays a heat map of adjusted RORs (aRORs) by sex and age group. The highest aROR was observed for hyperprolactinemia with risperidone (211.9, 95% CI 202.8–221.4). High aRORs were observed for extrapyramidal motor events, particularly dystonia with haloperidol (45.7, 95% CI 41.2–50.8) and akathisia with aripiprazole (45.2, 95% CI 41.9–48.8). Overall, clozapine had the lowest aRORs, except for anticholinergic syndrome, where the aROR was 4.1 (95% CI 2.7–6.3). The highest aRORs for anticholinergic syndrome were with olanzapine (16.2, 95% CI 12.0–21.8), followed by haloperidol, quetiapine, risperidone, and clozapine; aripiprazole had the lowest (2.4, 95% CI 1.2–4.6).

The risk for metabolic adverse events was highest with olanzapine, showing aRORs of 7.9 (95% CI 7.6–8.2) for hyperglycemia and 10.1 (95% CI 9.6–10.5) for weight gain. No metabolic risk signals were observed for clozapine.

For TdP/QT prolongation, haloperidol (6.8, 95% CI 5.7–8.0), quetiapine (6.3, 95% CI 5.9–6.8), and olanzapine (5.7, 95% CI 5.2–6.3) had the highest aRORs. Sedation‐related aRORs were elevated for all antipsychotics, with olanzapine (7.2, 95% CI 6.9–7.6), quetiapine (6.1, 95% CI 5.9–6.3), and haloperidol (5.3, 95% CI 4.7–5.9) being most prominent. Cognitive impairment risks were notable for all except clozapine. SIADH/hyponatremia was most strongly associated with olanzapine (4.7, 95% CI 4.2–5.2), risperidone (3.1, 95% CI 2.7–3.5), and haloperidol (3.1, 95% CI 2.5–4.0).

Hyperprolactinemia risk was exceptionally high for risperidone (211.9, 95% CI 202.8–221.4), with lower but significant signals for other antipsychotics except clozapine. Sexual dysfunction was observed with risperidone (2.2, 95% CI 2.0–2.5), quetiapine (2.3, 95% CI 2.1–2.5), olanzapine (2.6, 95% CI 2.3–2.9), and aripiprazole (3.2, 95% CI 2.9–3.5). Suicide risk was linked to all antipsychotics except clozapine, with the highest aRORs for quetiapine (6.5, 95% CI 6.3–6.7) and haloperisssdol (6.2, 95% CI 5.8–6.7).

Only clozapine had an association with agranulocytosis (2.0, 95% CI 1.9–2.2). No risk signal was observed for “accidents and injuries”, which includes incidents caused by altered perception, consciousness, attention, or behavior.

### 3.2 Age‐Related Differences in aRORs

**FIGURE 2:**
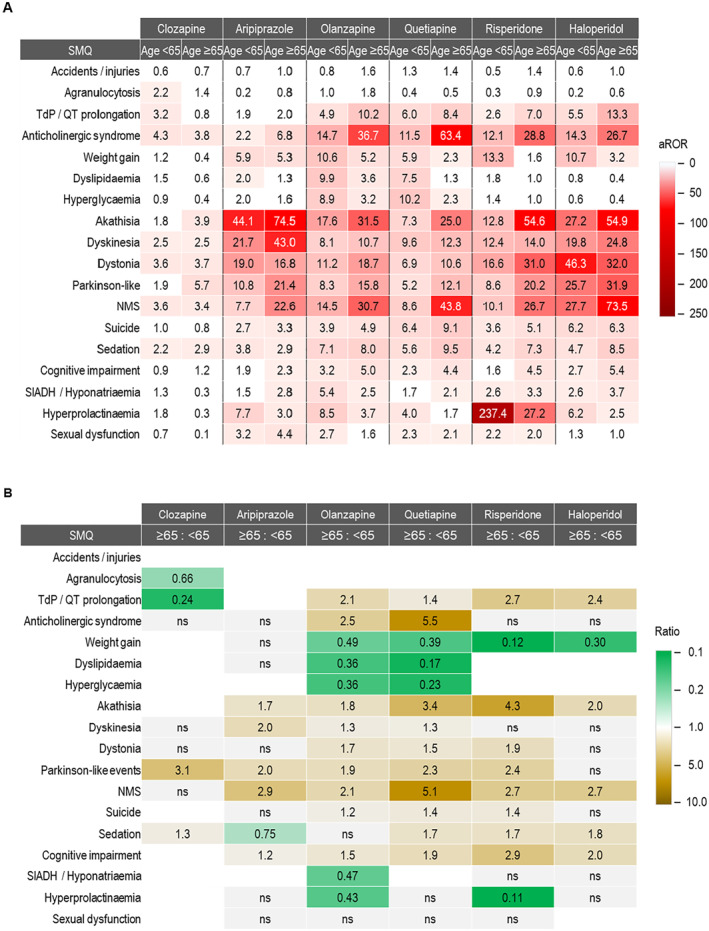
Heat maps displaying age‐specific, sex‐adjusted reported odds ratios (aRORs) and the ratios of these aRORs. (A) Heat map of age‐specific aRORs, and (B) heat map of the ratios of aRORs for older (≥ 65 years) versus younger (< 65 years) patients. Cells are left blank if aROR < 2. NMS, neuroleptic malignant syndrome; TdP, torsade de pointes; SIADH, syndrome of inappropriate antidiuretic hormone secretion; ns, not significant (Z statistics).

Figure 2 shows heat maps of aRORs (adjusted for sex) for patients aged ≥ 65 versus < 65 (A), along with the ratios of aRORs between the two age groups (B). Older patients (≥ 65) generally had a higher risk of adverse events related to extrapyramidal motor syndrome, neuroleptic malignant syndrome (NMS), sedation, and cognitive impairment, especially with quetiapine. For instance, older patients had 5.5 times higher odds of anticholinergic syndrome, 1.3 to 3.4 times higher odds of extrapyramidal motor disorders, and 5.1 times higher odds of NMS.

In contrast, younger patients (< 65) had higher odds of metabolic adverse events such as hyperglycemia, hyperlipidemia, and weight gain. Younger patients on olanzapine, quetiapine, risperidone, or haloperidol had 2.0 to 8.2 times higher odds of weight gain, and those on olanzapine and quetiapine had 2.8 and 5.7 times higher odds of hyperglycemia and dyslipidemia, respectively. The forest plots in Figure 3 display the reporting odds ratios (RORs) with 95% confidence intervals for each SMQ–drug pair, stratified by age and sex subgroups.

FIGURE 3Forest plots showing the reporting odds ratios (RORs) with 95% confidence intervals for each SMQ–drug pair, stratified by age and sex subgroups. NMS, neuroleptic malignant syndrome; SIADH, syndrome of inappropriate antidiuretic hormone secretion; TdP, torsade de pointes.

**Figure:**
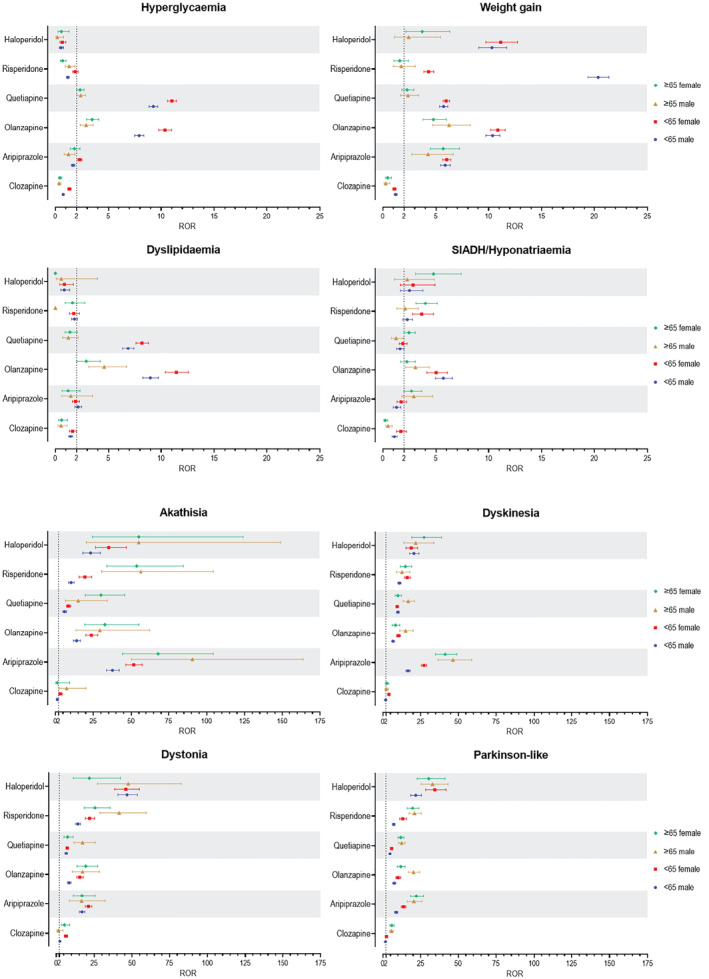

**Figure:**
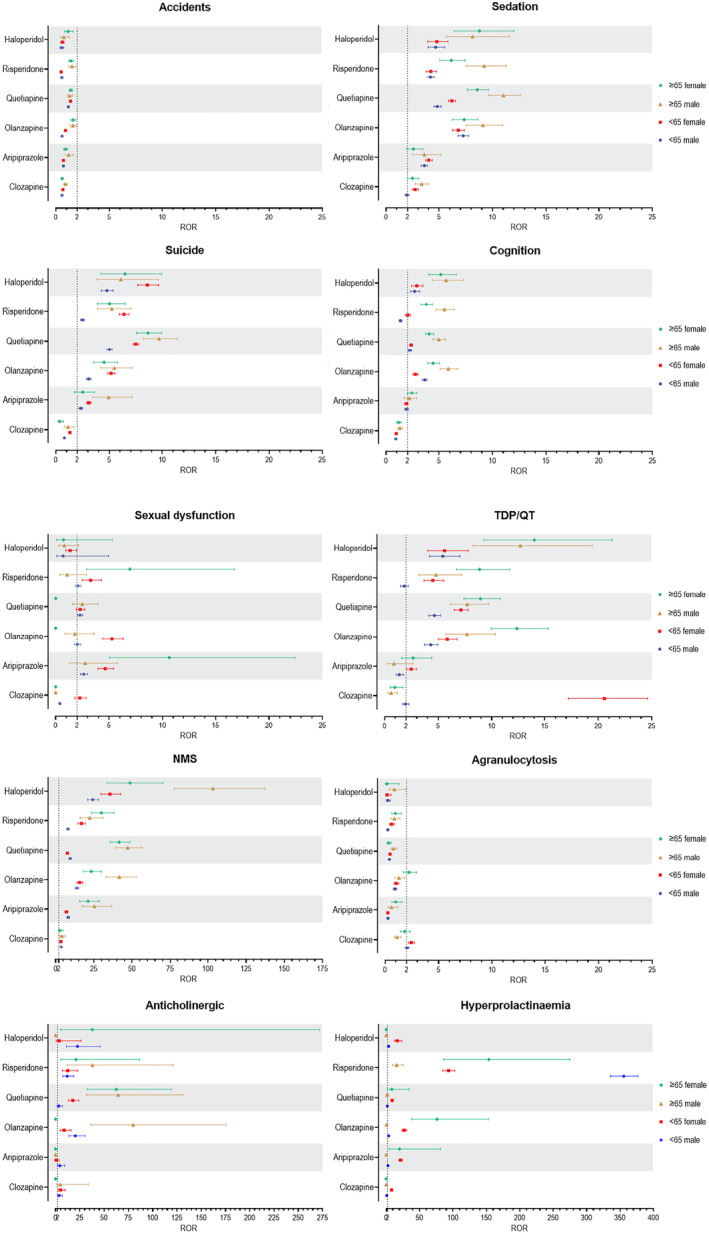

Age‐related differences were not consistent across all SMQs or antipsychotics. For example, no significant age‐related differences were observed for haloperidol in akathisia, dyskinesia, dystonia, or Parkinson‐like events, while quetiapine showed higher aRORs for these in older patients. Most drugs showed similar aRORs for anticholinergic syndrome across age groups, except quetiapine, which had a 5.5‐fold higher aROR in older adults. Antipsychotic drugs generally showed 1.4 to 2.7 times higher odds of QT prolongation and TdP in older adults, except aripiprazole, which showed no significant age effect, and clozapine, which had 4.2 times higher odds in younger patients.

### 3.3 Sex‐Related Differences in aRORs

**FIGURE 4:**
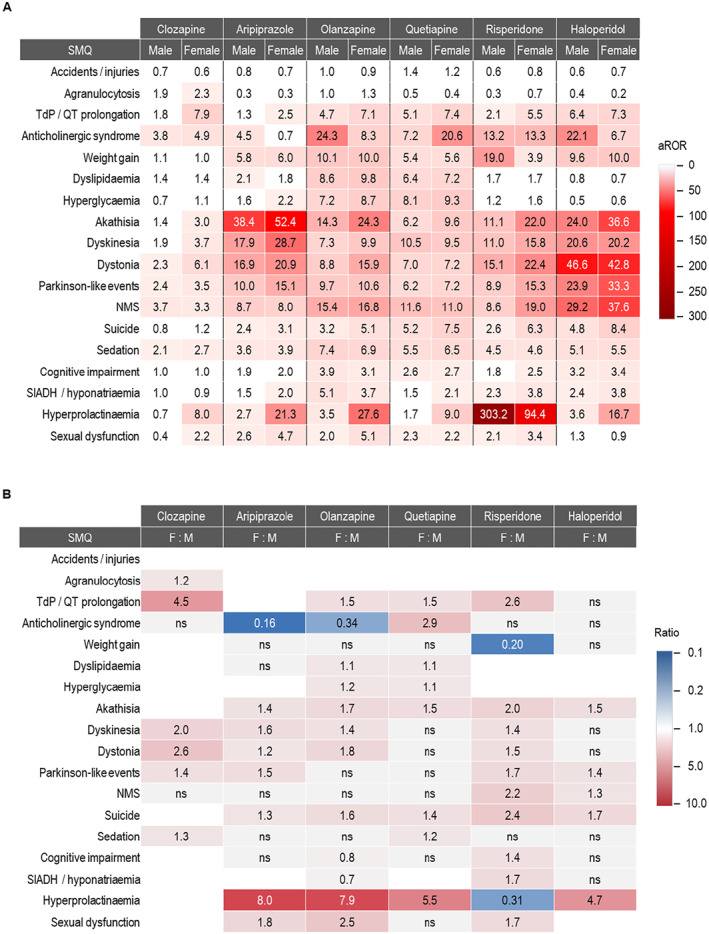
Heat maps displaying sex‐specific, age‐adjusted reported odds ratios (aRORs) and the ratios of these aRORs. (A) Heat map showing sex‐specific aRORs, and (B) ratios of aRORs for female versus male patients. Cells are left blank if aROR < 2. F, female; M, male; NMS, neuroleptic malignant syndrome; ns, not significant (Z statistics); SIADH, syndrome of inappropriate antidiuretic hormone secretion; TdP, torsade de pointes.

Figure 4 shows heat maps of aRORs (adjusted for age) for females versus males (A) and the ratios of aRORs between the two sex groups (B). Generally, aRORs were either similar or higher in women, with a few exceptions. For olanzapine, RORs for anticholinergic syndrome, cognitive impairment, and SIADH/hyponatremia were 2.9, 1.3, and 1.4 times higher in men, respectively. Similarly, for risperidone, RORs for hyperprolactinemia and weight gain were 3.2 and 4.9 times higher in men than in women, respectively. The forest plots in Figure 3 display the reporting odds ratios (RORs) with 95% confidence intervals for each SMQ–drug pair, stratified by sex and age subgroups.

The most significant sex differences were observed for hyperprolactinemia. Women on aripiprazole and olanzapine had 8.0 and 7.9 times higher odds of developing hyperprolactinemia than men, respectively. For quetiapine and haloperidol, the odds were 5.5 and 4.7 times higher in women. The only exception was risperidone, where men had 3.2 times higher odds of hyperprolactinemia than women.

**FIGURE 5:**
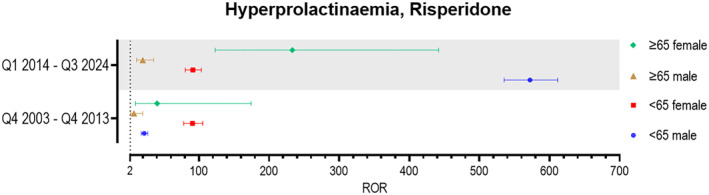
Forest plots showing the reporting odds ratios (RORs) with 95% confidence intervals for the SMQ hyperprolactinemia associated with risperidone as the primary suspect drug during the periods during the periods Q4 2003–Q4 2013 and Q1 2014–Q3 2024.

To further explore the unique pattern observed for risperidone—distinct from other antipsychotics—we conducted additional analyses. We found a sharp increase in the number of reported cases identifying risperidone as the primary suspect drug for hyperprolactinemia (SMQ) in men, rising from an average of 10 cases per year before 2014 to 398 cases per year from 2014 onward. In contrast, reports involving women showed only a modest increase, from an average of 22–33 cases per year. Based on this trend, we performed separate analyses for the periods before and after January 2014. Prior to 2014, a significant signal for risperidone‐associated hyperprolactinemia was observed, with a higher ROR in women than in men. However, after 2014, this pattern reversed, with a stronger signal observed in men (see Figure 5).

To assess the robustness of the analysis, we compared results based on “primary suspect” designations in FAERS case reports with an alternative approach that included all designation types (“primary suspect,” “secondary suspect,” “concomitant,” and “interacting”). Overall, the risk signals were largely consistent between the two approaches, with only a few exceptions (see Figure S1 in the Supplement). These exceptions primarily involved clozapine, for which risk signals were observed for certain SMQs and age/sex subgroups when all designation types were included, but not when the analysis was restricted to “primary suspect” cases.

## 4 Discussion

Recent studies have assessed antipsychotic adverse effects using systematic reviews and network meta‐analyses (NMA) of randomized controlled trials (RCTs) [16, 17]. However, NMAs have limitations, including study heterogeneity, which can bias comparisons, and the exclusion of certain populations, such as older adults, from RCTs, reducing the applicability of findings to real‐world settings. Observational studies, such as our study, analyzing real‐world data, provide important insights into the safety of antipsychotics in older patients and may aid in clinical decision‐making [18].

Several studies have used FAERS to identify adverse event signals associated with antipsychotic drugs, typically focusing on specific agents (e.g., lumateperone, asenapine) or specific adverse events (e.g., venous thromboembolism, cutaneous toxicity, hepatotoxicity, hyperprolactinemia, eye disorders), and most have not examined differences by sex or between geriatric and non‐geriatric populations [19, 20, 21, 22, 23, 24, 25, 26]. To our knowledge, only one study has compared adverse event profiles across younger (≤ 65 years) and geriatric (> 65 years) groups [27]. It reported age‐related differences in the frequency and types of AEs for several antipsychotics, including aripiprazole, clozapine, haloperidol, olanzapine, quetiapine, risperidone, and ziprasidone—for instance, diabetes was most frequently reported in adults, behavioral issues in children, and neurological symptoms in older adults [27]. However, a key methodological limitation was its reliance on raw reporting frequencies rather than disproportionality analysis, the standard approach for signal detection in FAERS. Our study is the first to compare the adverse effects of commonly prescribed antipsychotics while examining age differences using the FAERS pharmacovigilance database. By employing a case‐control design, we calculated adjusted reporting odds ratios (aRORs) for quantitative comparisons [14]. Our findings show that age and sex significantly affect the adverse effect profiles of these medications, highlighting the importance of a more personalized treatment approach to reduce risks and improve therapeutic outcomes.

### 4.1 Age‐Related Adverse Event Profiles

Our study highlights significant age‐related differences in the adverse events of antipsychotics, particularly showing that older patients are more sensitive to certain reactions. Those aged 65 and older were particularly vulnerable to extrapyramidal symptoms like parkinsonism, dystonia, and dyskinesia, especially with haloperidol and aripiprazole. These findings align with evidence that age‐related changes in neurotransmission and receptor sensitivity increase susceptibility to such effects [13, 28, 29].

Cardiovascular risks, such as TdP and QT prolongation, were found to be significantly higher in older adults, with haloperidol posing the greatest risk in our study, followed by olanzapine and quetiapine. Although previous studies have associated antipsychotics with QT prolongation, they often failed to account for differences in age or specific drugs [30, 31]. Notably, a meta‐regression analysis revealed that older adults generally have a higher prevalence of QTc prolongation, but it did not evaluate individual antipsychotics [30]. Similarly, a separate meta‐analysis highlighted the significant risk of QTc prolongation with haloperidol, particularly when administered intravenously at high doses. This analysis also identified quetiapine and amisulpride as notable risks, though it did not examine the influence of age [31]. Our findings help fill this gap by providing detailed odds of adverse cardiac events associated with individual antipsychotics in older versus younger adults. These results align with the 2023 AGS Beers Criteria, which advise caution when prescribing antipsychotics to older adults due to their increased cardiac risks [11].

In our study, risperidone and olanzapine showed the highest odds of weight gain, followed by haloperidol and quetiapine. These findings align with a meta‐analysis of RCTs that identified asenapine and olanzapine as having the greatest risk of significant weight gain compared to placebo, followed by risperidone and extended‐release quetiapine [32]. While previous research on the effects of age on the metabolic side effects of antipsychotics has largely focused on children and adolescents [33], data on these risks in older adults remain scarce. Our study addresses this gap, revealing that the odds of weight gain and metabolic abnormalities such as hyperglycemia, and dyslipidemia are generally lower in geriatric patients compared to younger adults.

Sedation was significantly linked to all the antipsychotic drugs studied, with the highest aROR ranging from 7.2 to 5.3 for olanzapine, quetiapine, and haloperidol. A network meta‐analysis of RCTs revealed that these drugs had notably higher relative risk ratios for sedation compared to placebo: 3.3 for quetiapine, 3.0 for clozapine, 2.2 for olanzapine, and 1.9 for haloperidol [16]. Our findings are the first to demonstrate and quantify a greater sedation risk in geriatric populations compared to younger adults. This supports recommendations like the PRISCUS 2.0 list, which classifies these antipsychotics as potentially inappropriate for older adults due to sedation‐related fall and fracture risks [12].

The MedDRA‐recommended SMQ “accidents and injuries” for studying falls in older adults did not detect an increased fall risk from the antipsychotics studied. However, falls are often multifactorial, involving poor strength, balance, visual or cognitive impairments, frailty, and the use of fall risk‐increasing drugs (e.g., antipsychotics, antihypertensives, antiarrhythmics, anticholinergics, antihistamines, sedatives, antidepressants, and opioids). In patients with polypharmacy, attributing falls to a single drug is challenging, which may explain the lack of a clear safety signal. A systematic review further suggests that deprescribing fall risk‐increasing drugs alone may have a limited impact on reducing falls when used as the sole preventive strategy [34]. The AGS Beers Criteria recommend avoiding concurrent use of three or more CNS‐active agents (e.g., antiepileptics, antidepressants, antipsychotics, benzodiazepines, nonbenzodiazepine receptor agonists, hypnotics, opioids, and muscle relaxants) due to an increased risk of falls and fractures [11].

Most individuals with schizophrenia experience cognitive deficits, and research indicates that second‐generation antipsychotics can improve both cognition and general symptoms of the disorder [35]. However, long‐term use, particularly of antipsychotics with anticholinergic properties, may lead to cognitive decline, especially in older adults [36, 37]. Our study identified cognitive impairment signals for all investigated antipsychotics except clozapine, with these effects being more pronounced in patients over 65. Interestingly, the signals did not strongly correlate with cholinergic receptor blockade or anticholinergic burden (ACB) scores [36]. While clozapine, olanzapine, and quetiapine have high ACB scores, risperidone, aripiprazole, and haloperidol have low scores.

Our study highlights that older adults are significantly more vulnerable to adverse effects, particularly extrapyramidal, cardiovascular, endocrine, and sedative reactions. These findings, along with recommendations from the AGS Beers Criteria and PRISCUS 2.0 list [11, 12], underscore the importance of carefully assessing risks and benefits before prescribing antipsychotics to older patients. Safer alternatives should be prioritized whenever feasible [11, 12].

### 4.2 Sex‐Related Adverse Event Profiles

Our analysis reveals significant sex‐specific differences in susceptibility to antipsychotic adverse events, with women generally at higher odds than men. This supports earlier findings by Barbui et al., who identified sex as a key factor in antipsychotic tolerability 2 decades ago [38]. Unlike their study, which did not differentiate between specific drugs, our research examined drug‐specific variations in side effect profiles.

One notable example is hyperprolactinemia, a common endocrine disorder linked to antipsychotics [39, 40]. While many first‐ and second‐generation antipsychotics can induce hyperprolactinemia, risperidone is particularly potent due to its strong antagonism of dopamine D2 receptors and its active metabolite, paliperidone. In our study, the age‐ and sex‐adjusted ROR for hyperprolactinemia with risperidone was exceptionally high at 212, compared to 3.9 to 8.2 for other antipsychotics.

A study of serum prolactin levels in schizophrenia found that while baseline prolactin levels were similar in unmedicated men and women, the overall prolactin response to antipsychotics was greater in women [41]. Our findings support this, showing that women report hyperprolactinemia significantly more often. Initial analyses suggested that risperidone might be an exception, with higher reporting odds ratios (RORs) in men than in women. However, further analysis indicates that this apparent male predominance was likely driven by lawsuits against the manufacturer of risperidone related to gynecomastia in young men, along with increased media attention beginning in 2014 [42]. This likely contributed to a sharp rise in gynecomastia reports from that time onward, leading to elevated RORs for the SMQ for hyperprolactinemia in our analysis.

Konarzewska et al. compared the effects of risperidone and olanzapine on the hypothalamo‐pituitary‐gonadal axis in men and found risperidone caused a greater prolactin elevation and greater disruptions in testosterone and follicle‐stimulating hormone levels than olanzapine. They also observed higher rates of sexual dysfunction and treatment discontinuation in male risperidone users compared to those on olanzapine [43]. Hyperprolactinemia symptoms are sex‐specific: women may experience menstrual irregularities or amenorrhea, while men may present with gynecomastia, erectile dysfunction, and impaired sperm production [44].

Sexual dysfunction is a significant side effect of antipsychotics, driven by mechanisms such as dopamine D2 receptor blockade, hyperprolactinemia, alpha‐1 adrenoceptor antagonism, cholinergic receptor antagonism, and sedation [45]. A recent meta‐analysis found a global prevalence of 55.7% (95% CI, 48.1–63.1) in men and 60.0% (95% CI, 48.0–70.8) in women [46]. In our study, we found no significant sex differences in sexual dysfunction with clozapine, quetiapine, or haloperidol. However, with aripiprazole, olanzapine, and risperidone, women had 1.7 to 2.5 times higher odds of experiencing sexual dysfunction compared to men.

In line with our findings, data from the large observational pharmacovigilance program AMSP (Arzneimittelsicherheit in der Psychiatrie), conducted in psychiatric settings across German‐speaking countries, also highlight sex‐ and age‐related patterns in adverse drug reactions (ADRs) to psychotropic medications. For example, Seifert et al. examined the risk of psychotropic drug‐related ADRs in psychiatric inpatients aged ≥ 65 years versus < 65 years (*n* = 462,661) [47]. Overall, older adults using antipsychotic drugs (APDs) had a significantly lower risk of ADRs compared to younger patients. However, low‐potency first‐generation antipsychotics (FGAs) were linked to a higher ADR risk in older adults, while high‐potency FGAs showed no consistent age‐related differences. Among second‐generation antipsychotics (SGAs), older adults had a lower overall ADR risk, except for clozapine (higher risk) and risperidone and olanzapine (lower risk). However, the study did not provide a detailed analysis of individual antipsychotic ADR profiles by age group, limiting direct comparison with our FAERS‐based pharmacovigilance analysis [47]. Glocker et al. (2021) reported on the incidence of galactorrhea due to elevated prolactin levels during antipsychotic treatment, identifying significant sex‐ and age‐related differences within the AMSP cohort [48]. Among 320,383 inpatients (175,884 women) treated with antipsychotics and recorded in the AMSP database between 1993 and 2015, 170 cases of galactorrhea were reported. The highest incidence occurred in women aged 16–30 years (3.81 per 1000). Risperidone (0.19%), amisulpride (0.48%), and olanzapine (0.05%) were most frequently identified as sole causative agents [48]. Schneider et al. (2020) demonstrated that both age and sex are key determinants in the occurrence of severe weight gain among psychiatric inpatients [49]. These real‐world data further underscore the importance of demographic factors in the risk profiling of antipsychotic‐induced side effects. Our findings highlight that signals for adverse events of antipsychotics are significantly influenced by sex. Yet, clinical trials and guidelines, such as the Practice Guideline for the Treatment of Patients with Schizophrenia, often treat patients as a homogeneous group, overlooking the notable sex‐based differences in tolerability of antipsychotics [50].

### 4.3 Strengths and Limitations

A key strength of our study is the use of the FAERS database, which includes data from a large, diverse patient population. This allowed us to investigate age‐specific adverse events, particularly in geriatric patients often excluded from RCTs. Analyzing 2 decades of data, we identified patterns in susceptibility to antipsychotic‐related adverse events across age groups and sexes, helping to tailor treatment strategies and support clinical decision‐making. However, there are limitations.

FAERS reports are mandatory for manufacturers but voluntary for healthcare professionals, leading to reduced reporting as side effects become more widely known. This may explain the lack of a risk signal for clozapine's metabolic side effects, such as weight gain. Furthermore, FAERS reports often lack key clinical details like dosage, concurrent medications, or the severity of adverse effects, which can impact result interpretation.

Another consideration is that adverse event report volumes may vary across antipsychotics due to differences in prescribing frequency. While this affects absolute report counts, our use of Reporting Odds Ratios (RORs)—which compare the odds of a specific event for a drug versus all others—helps control for such variation. However, as FAERS lacks reliable exposure data, RORs reflect reporting patterns rather than true incidence rates and should be interpreted with this limitation in mind.

This analysis used a dichotomous age grouping (< 65 vs. ≥ 65 years), which may introduce heterogeneity within both age categories. Each group encompasses a wide range of developmental and clinical stages that may differ in their pharmacokinetic profiles and susceptibility to antipsychotic‐related adverse events. For example, the < 65 category includes individuals ranging from pediatric and adolescent to middle‐aged adults, while the ≥ 65 category includes both early (65–74 years) and late (≥ 75 years) geriatric populations, who may differ significantly in terms of frailty and comorbidities. This limitation should be considered when interpreting subgroup findings, and future studies with larger datasets may benefit from more granular age stratification.

While our findings highlight age‐related differences in adverse event (AE) profiles with antipsychotic use, additional factors such as polypharmacy, treatment indication, reporter type, prescribing rates, dosage, dosage form, and dose titration may also influence AE patterns. However, further subgroup analyses were not feasible due to limited case numbers and incomplete data within FAERS, which would have undermined statistical power and reliability. We acknowledge these limitations and encourage future research using larger, more detailed datasets to explore these factors further.

## 5 Conclusions

Our study shows that age and gender significantly affect the adverse event profiles of antipsychotics. Older patients are more vulnerable to cardiovascular, extrapyramidal, and sedative effects, while women are more prone to endocrine and metabolic issues. These findings underscore the need for individualized treatment plans that account for age‐ and sex‐specific differences to reduce adverse events and improve outcomes. A personalized approach is essential for enhancing patient safety and quality of life. Further research is needed to better understand these differences and refine treatment guidelines.

## Conflicts of Interest

The authors declare no conflicts of interest.

## Supporting information


**Figure S1**: Sensitivity analysis comparing results based on “primary suspect” designations in FAERS case reports with an alternative approach that included all designation types (“primary suspect,” “secondary suspect,” “concomitant,” and “interacting”).

## Data Availability

The datasets used and/or analyzed during the current study are available from the corresponding author upon reasonable request.
