# Supplementary material for: Age and Sex Differences in Adverse Events Associated With Antipsychotics: An Analysis of the FDA Adverse Events Database
Source: Int J Geriatr Psychiatry. 2025 Aug 15;40(8):e70142. doi: 10.1002/gps.70142 (PMC12356759; doi:10.1002/gps.70142)
Supplement: Supplementary file 1 — Figure S1: Sensitivity analysis comparing results based on “primary suspect” designations in FAERS case reports with an alternative approach that included all designation types (“primary suspect,” “secondary suspect,” “concomitant,” and “interacting”). [file GPS-40-e70142-s001.docx]

**SUPPORTING MATERIAL to**

**Age and Sex Differences in Adverse Events Associated with Antipsychotics: An Analysis of the FDA Adverse Events Database**

Tabea Ramin^1, §^, Jens-Uwe Peter^1, §^, Michael Schneider^2, 3^, Volker Dahling^2, 3^, Oliver Zolk^1, 3^

^1^ Institute for Clinical Pharmacology, Immanuel Hospital Rüdersdorf, Brandenburg Medical School Theodor Fontane, Seebad 82/83, 15562 Rüdersdorf, Germany

^2^ Center for Mental Health, Immanuel Hospital Rüdersdorf, Brandenburg Medical School Theodor Fontane, Seebad 82/83, 15562 Rüdersdorf, Germany

^3^ Faculty of Health Sciences, joint Faculty of the University of Potsdam, the Brandenburg Medical School Theodor Fontane and the Brandenburg University of Technology Cottbus – Senftenberg, Fehrbelliner Straße 38, 16816 Neuruppin, Germany

^§^ shared first authorship

Supplementary Figure S1:

Sensitivity analysis.


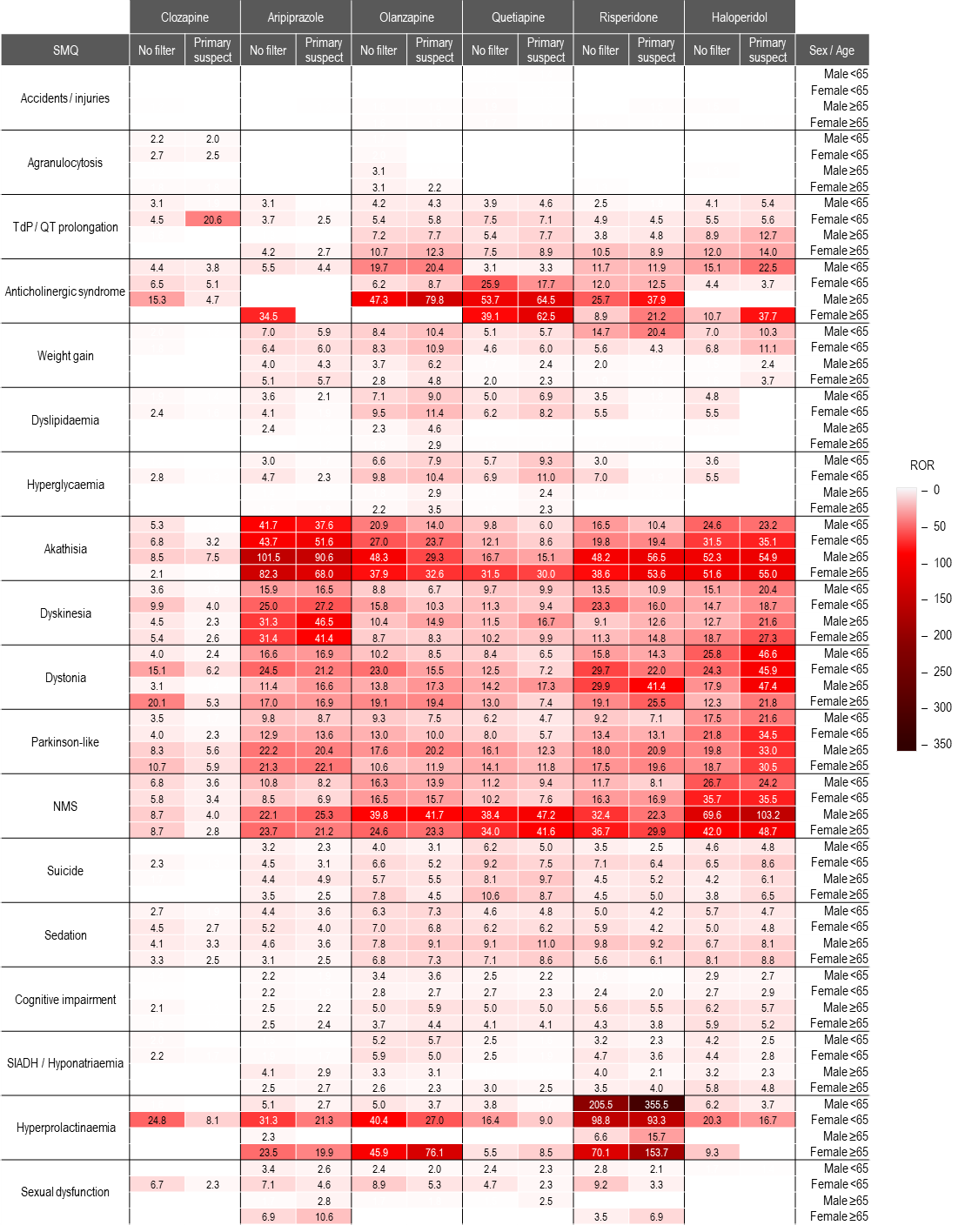


**FIGURE S1**

Sensitivity analysis comparing results based on “primary suspect” designations in FAERS case reports with an alternative approach that included all designation types (“primary suspect,” “secondary suspect,” “concomitant,” and “interacting”), referred to as “no filter” in the figure. For each SMQ–drug pair and age/sex subgroup—whenever a risk signal was detected—ROR values are presented and visualized using a heat map color code. Abbreviations: SMQ, standardized MedDRA query; NMS, neuroleptic malignant syndrome; TdP, torsades de pointes; SIADH, syndrome of inappropriate antidiuretic hormone secretion.
